# An Examination of a New Psychometric Method for Optimizing Multi-Faceted Assessment Instruments in the Context of Trait Emotional Intelligence

**DOI:** 10.1002/per.1976

**Published:** 2014-11-05

**Authors:** A B Siegling, K V Petrides, Khatuna Martskvishvili

**Affiliations:** 1 London Psychometric Laboratory, University College LondonLondon, UK; 2 Tbilisi State UniversityTbilisi, Georgia

**Keywords:** scale construction and development, facets, psychometrics, assessment, trait emotional self-efficacy, TEIQue

## Abstract

Driven by the challenge of representing and measuring psychological attributes, this article outlines a psychometric method aimed at identifying problem facets. The method, which integrates theoretical and empirical steps, is applied in the context of the construct of trait emotional intelligence (trait EI), using data from six different samples (N = 1284) collected across Europe. Alternative representations of the trait EI variance, derived from the outcome variables used in previous validation studies of the Trait Emotional Intelligence Questionnaire, were regressed on the 15 trait EI facets using the stepwise method. The analyses revealed five facets, which did not occupy unique construct variance in any of the six samples. As expected, a composite of the remaining 10 facets consistently showed greater construct validity than the original 15-facet composite. Implications for construct and scale development are discussed, and directions for further validation of the method and for its application to other constructs are provided. © 2014 The Authors. European Journal of Personality published by John Wiley & Sons Ltd on behalf of European Association of Personality Psychology.

Examining the literature of an individual-differences construct, one often finds a diversity of measures, with an overall abundance of facets. Even individual measures composed of a fairly large number of facets are quite common. In some cases, the arrays of facets used to represent the same construct diverge considerably (in quantity and/or types), and correlations between their composites are weak or moderate (e.g. Baer, Smith, Hopkins, Krietemeyer, & Toney, 2006; Brackett & Mayer, 2003). It is then difficult to accept that all measures reflect the same underlying attribute to a similar degree. This rather messy state reflects the lack of adequate criteria for defining psychological constructs, which are only indirectly inferable and measurable (Cronbach & Meehl, 1955). Thus, researchers have noted that there is considerable uncertainty in determining the set of facets and models from which the composite representative of the targeted attribute can be derived (e.g. Petrides & Furnham, 2001).

The present article describes and applies a new psychometric method for developing and optimizing multi-faceted measurement instruments. Because scale development goes hand-in-hand with the development of construct representations (e.g. structural models), it also has implications for the latter. The method is intended to supplement the contemporary theoretical and empirical approaches to scale construction, by targeting ‘problem’ facets detrimental to construct validity. It thereby aims to minimize the plethora of facets through which constructs are often represented. The basic principle of the method is to identify problem facets based on their inability to occupy a unique part of the target construct's variance. It uses an alternative representation of the construct to assess whether a measure's facets fulfil this general criterion.

Prior to describing the method in detail, it is necessary to specify its unique focus and explain how it supplements existing test construction methods. We then proceed with a brief review of the construct of trait emotional intelligence (trait EI), on which the method will be applied in the present investigation. Similar to definitions commonly used in the literature (Costa & McCrae, 1995; Smith, Fischer, & Fister, 2003), we use the term *facet* to refer to a variable representing a narrow and highly homogenous subset of affective, behavioural, or cognitive tendencies associated with a given construct. Facets are interrelated and define the hypothetical domain of a construct; their common variance is conceptualized as representing the construct of interest. We use the term *factor* to designate a variable that subsumes the common, construct-related variance of several facets. Factors provide a mid-level between facets and the latent construct, serving to organize the facets into subcategories and providing the basis for subscales.

## Rationale and focus: Redundant and extraneous facets

The psychometric literatures of numerous constructs suggest that the contemporary scale-construction approaches lack efficacy in screening out a considerable number of problem facets. This is not particularly surprising, because their primary goal is to identify relevant content and build structural models, rather than to optimize and refine construct representations. In short, we argue that the contemporary psychometric approaches lack utility in identifying problem facets and thereby contribute to the inflation in the number of facets often seen in the literature. Further, we are convinced that this limitation plays a salient role in the diversification of measures.

### Defining problem facets

We specify here three criteria a variable should meet in order to qualify as a useful facet of a higher-order construct. First, facets must tap into a homogenous set of psychological processes, situated at the same ontological level. Essentially, this means that a facet represents a set of proximate manifestations of the construct, rather than some distant outcome, indirectly associated with the construct (e.g. number of friends or romantic partners, highest level of education achieved, or age of death), or even an antecedent of the construct (e.g. parenting style). Second, a facet should share a non-negligible amount of variance with the other facets. Modest correlations between facets, or weak loadings of individual facets on the latent composite, may be due to untargeted sources, such as other constructs or response biases. However, although often taken as such, the common variance is insufficient as the sole empirical criterion for the validity of facets. A third criterion is that a facet should occupy a unique portion of the variance attributed to the construct it is theorized to represent (i.e. common variance not covered by other facets). This last criterion is the main focus of the method presented here.

**Figure 1:**
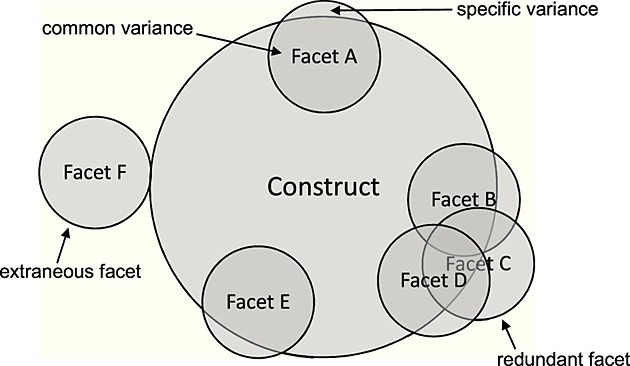
Illustration of redundant and extraneous facets with respect to their component (i.e. common and specific) variance.

As regards the second and third criteria earlier, two types of problem facets can be operationally defined. We refer to them as *extraneous* and *redundant* facets (hereafter abbreviated as ET and RD facets, respectively). The best way to describe these facets is with respect to their component variance, as graphically illustrated in Figure1. Facets can have two types of variance: reliable common variance, which is due to the target construct and shared with the other facets, and reliable specific variance, which is unrelated to the target construct (Smith et al., 2003). ET facets have no common variance at all (i.e. variance due to the target construct); their variance is due to dimensions other than the one reflecting the target construct, thus likely violating the second criterion. As indicated, however, ET facets may still share variance with valid facets, because of measurement bias or dimensions other than the target construct. Although RD facets have common (construct) variance, this variance is more efficiently covered by at least one other. Therefore, RD facets do not occupy ‘unique common variance’ and do not add to the comprehensive representation of the construct (Criterion 3).

Both these two types of problem facet compromise the construct validity of a model or set of facets. RD facets lead to an unbalanced representation of the target construct's variance by over-representing some of its manifestations, while ET facets result in representations that extend beyond the target construct's boundaries, representing expressions of other, non-targeted dimensions. At the empirical level, both are prone to compromising the validity of the global composite derived from the facet scores. Neither is uniquely representative of the target construct and, hence, unlikely to occupy a distinctive portion of its variance vis-à-vis the other facets. When combined into a global composite, the effects of predictive facets are averaged out with those of the non-predictive facets (Smith et al., 2003). Consequently, the correlations of their composite with construct-relevant outcomes are lower than those of a composite encompassing exclusively predictive facets. Because ET facets stretch the variance of the composite thought to represent the target construct into other dimensions, they also impose construct-unrelated variance on the composite.

### Limitations of contemporary psychometric approaches

The existing methods have been classified as the deductive, inductive, and external approaches (Burisch, 1984) or, alternatively, as the rational–theoretical, internal consistency, and criterion-keying approaches, respectively (Burisch, 1984; Simms & Watson, 2007). Although the rational–theoretical approach encompasses the largest number of specific methods (e.g. content analysis, focus groups, and evidence-oriented methods), coming up with an optimal representation of the construct based on theory and reasoning alone is virtually impossible. Items or facets that appear to be conceptually relevant may not represent variance attributable to the target construct. Furthermore, as discussed, even thematically and empirically related facets may not represent a unique aspect of the construct relative to the other facets within the model.

The internal consistency approach subsumes the range of variations and applications of factor analysis. However, this approach cannot identify RD facets, because it does not reveal whether a facet occupies a unique part of the construct variance not already covered by one or more of the other facets. In fact, RD facets are likely to have inflated factor loadings, leading to overrepresentations of certain manifestations of the construct and their variance within the total composite. Further, although this approach may reveal many ET facets, it cannot identify them reliably. Factor loadings depend on the facets in the model being tested. If a set of facets represents the construct weakly, ET facets are more likely to load on the latent composite. Also, ET facets are particularly likely to be retained where low cut-offs are used, which is a problem given that there are no agreed-on guidelines regarding the magnitude of factor loadings and communalities at which one should retain facets (Gignac, 2009).

In contrast to the internal consistency approach, in which items or facets are selected based on their interrelationships, criterion-keying selects variables based on their ability to predict relevant external criteria. A variable's predictive ability has relevance for the identification of RD and ET facets, as these should not occupy any unique variance linked to the target construct. However, a widely discussed shortcoming of this approach is the lack of a rational–theoretical component, because test items are selected from large item pools based on their predictive ability alone. Moreover, criterion-keying is restricted to attributes for which people at the low or high extreme can be identified fairly objectively (e.g. extraverts and introverts, narcissists, and people identified as having a particular disorder). For many constructs, it is difficult to classify individuals unambiguously, because there is no shared agreement of how people at the extremes are like, which relates back to the conceptual ambiguity of these constructs.

Variants of these traditional approaches or altogether different approaches focused on either construct testing or scale development have emerged in more recent years (Chen, Hayes, Carver, Laurenceau, & Zhang, 2012; Costa & McCrae, 1995; Hull, Lehn, & Tedlie, 1991; Smith et al., 2003). However, none of these addresses the problem of identifying RD and ET facets, which is the focus of the proposed method for optimizing assessment instruments outlined in this article.

## Description of new method

The psychometric method we propose here is intended to complement the existing scale-construction approaches, by helping to identify RD and ET facets. It is, thus, especially useful if one deals with ‘fuzzy’ constructs that lack consensual definitions. Presently divided into five broad steps, the method seeks to identify RD and ET facets based on their inability to occupy a unique part of the target construct's variance. As discussed, the common, construct-based variance of RD facets is already occupied by other facets, whereas ET facets do not overlap with the target construct. Consequently, both types of facet compromise, rather than enhance, the representation of the construct.

A basic premise of the method is that a variable representing the construct variance comprehensively can be derived from a source other than the construct's measurement vehicle. If such a variable can be extracted, it could be used as a benchmark to examine whether each of the hypothetical facets occupies a unique portion of the construct variance. Of course, sufficiently broad variables needed to represent the variance of most constructs do not pre-exist (Epstein, 1984). Individual outcome variables that are theoretically influenced by the target construct and commonly used to assess its criterion validity are unlikely to reflect its entire impact comprehensively. Moreover, they cannot be expected to represent the construct variance exclusively, and therefore, using multiple individual outcomes for the purpose of representing the construct would be no reasonable solution. Because of the specific variance that these criteria would bring into the equation, there would be an increased chance of seeing predictive effects of ET facets and, to a lesser extent, RD facets.

### Step 1

While using individual or multiple validation criteria is not instrumental for identifying RD and ET facets, a single variable that is representative of the target construct's variance should be defined by the shared variance of construct-relevant outcomes. Using latent composites of these outcome variables therefore appears to be a reasonable and practical solution to capturing the variance of a given construct comprehensively (hereafter, we use the term *outcome-based composite* to refer to variables representing the shared variance of construct-relevant outcomes). This composite can then be used to assess whether each of the hypothetical facets occupies unique construct variance. Thus, Step 1 is to obtain a comprehensive sample of construct-relevant outcomes with common-variance representative of the target construct. Naturally, Step 1 also involves administering the chosen set of outcomes along with a comprehensive and multi-faceted measure of the target construct to multiple samples.

Selecting outcome variables has a strong theoretical component, involving a systematic sampling process. Various approaches to selecting comprehensive sets of outcome variables are conceivable, although in general, it seems safest to rely on proximate outcomes (i.e. variables representing affect, behaviours, cognition, and desires) that share the general theme of the construct and correlate in the expected direction with it. More indirectly related outcomes increase the chances of significant incremental effects of ET facets.

While it may be impractical to administer a representative sample of measures to a single sample of participants, it would be legitimate to spread out the measures across samples to ensure that all parts of the construct variance are represented. The number of measures per sample would then depend on the total number of measures needed to represent the construct variance and on how many measures one can reasonably administer to each sample without compromising the validity of the responses. Ideally, one would randomly assign outcomes corresponding to each empirically or theoretically derived higher-order factor across samples to ascertain that their common variance is representative of the target construct.

### Step 2

In Step 2, one extracts the first principal component from the chosen set of criteria, because it is, in theory, the one that is representative of the target construct's variance. Divergent outcome variables, specifically those that have low loadings on the first principal component and that mostly vary because of sources other than the target construct, can be readily identified and excluded. The method can thereby account for and, to some extent, resolve inconsistencies in researchers' conceptualizations of the target construct and in the outcomes they deem relevant.

### Step 3

Step 3 of the method examines whether each of the facets occupies a significant portion of variance in the derived outcome-based composite. Facets that consistently fail to account for variance in this composite are likely to be redundant or extraneous and should be excluded from the set of facets used to represent the construct. The most straightforward statistical procedure for this purpose is to regress the outcome-based composite on the theoretical set of facets, using statistical regression (also referred to as the stepwise method) to remove facets, although starting with all hypothetical facets at the initial step. Stepwise regression is the appropriate algorithm in this instance, as it both removes and adds predictors. Facets will be removed from the analysis successively if they do not explain unique variance in the criterion. In this process, RD and ET facets may initially suppress the (significant) effects of valid facets and lead to their removal at initial steps. Yet, the stepwise method re-enters facets removed from the analysis at preceding steps if they gain their significant explanatory effect at later steps (i.e. upon removal of problem facets with suppressor effects).

High intercorrelations among predictors are generally considered problematic, because they can compromise the explanatory effects of individual predictors (Pedhazur, 1997). However, in conjunction with the systematic removal of facets via stepwise regression, the method advanced here capitalizes on this principle in order to identify RD facets. Essentially, it means that highly correlated predictors are likely to explain the same variance in the criterion, rendering some as redundant. The method is sufficient in identifying facets that do not occupy a significant part of the construct variance represented by the outcome-based composite (ET facets should not occupy any construct variance, irrespective of the presence of other facets).

### Step 4

Step 4 of the method involves a comparison of the composite excluding any facets that were consistently non-predictive of the outcome-based composites (i.e. ET and RD facets) against the original composite comprising all facets. These two composites are compared in their degree of convergence with the outcome-based composite derived at Step 2. Using a composite of all facets averages predictive and non-predictive facets, and the correlation of this composite with the outcome-based composite should be weaker than that of a composite encompassing predictive facets only (see Smith et al., 2003, for a more detailed discussion of this effect).

### Step 5

Zero-order correlations of the identified non-predictive facets with the revised composite can be examined in a final step (Step 5) to distinguish between RD and ET facets. RD facets should show substantial zero-order correlations with this composite, whereas ET facets should not.

## Trait emotional intelligence

A construct of contemporary interest that illustrates the challenge of representing constructs is EI. Much has been said about what constitutes EI, as is apparent from the diversity of EI models and operationalizations. The divergence of research into the two increasingly distinct subareas of trait EI and ability EI has brought some structure into the field. Petrides and Furnham (2001) pointed to the fundamentally distinct nature of constructs based on typical performance, the predominant measurement method in the EI literature, as compared with those that are based on maximum performance. But even when taking the split between typical-performance and maximum-performance measures into consideration, substantial discrepancies in how the construct is represented via structural models and arrays of facets remain across measures (cf. Dulewicz, Higgs, & Slaski, 2003; Jordan, Lawrence, 2009; Petrides & Furnham, 2001; Salovey, Mayer, Goldman, Turvey, & Palfai, 1995; Schutte et al., 1998; Tapia & Marsh, 2006; Tett, Fox, & Wang, 2005); the construct boundaries are far from agreed upon.

Trait EI has provided a framework for reconceptualizing self-report measures of EI initially supposed to assess cognitive emotional abilities, which they are hardly able to measure (Freudenthaler & Neubauer, 2007; Paulhus, Lysy, & Yik, 1998). However, the distinction of ability and trait EI goes beyond mere operational differences in response format. For example, self-report measures based on Mayer and Salovey's (1997) four-branch ability EI model do not seem to measure trait EI comprehensively, as evidenced by their relatively weak construct validity compared with instruments developed to measure trait EI specifically (Gardner & Qualter, 2010; Martins, Ramalho, & Morin, 2010). By definition, trait EI refers to a compound trait located at the lower levels of personality hierarchies that integrates the affective aspects of personality (Petrides, Pita, & Kokkinaki, 2007); it does not encompass emotion-related skills or abilities.

Trait EI is also conceptually distinct from the construct of social intelligence, irrespective of the method of measurement and conceptualization of trait versus ability. Whereas the former concerns primarily emotional aspects of personality, the latter reflects how people interact with others (e.g. Petrides, Mason, & Sevdalis, 2011). Of course, this does not preclude overlap in their sets of facets, because many specific attributes integrate social and emotional qualities (e.g. aggression, assertiveness, and empathy) and, thus, may be linked to both constructs. The key point is that these abstract and difficult-to-define constructs are fundamentally distinct in their core. One would find considerably more emotional/affective facets within a measure of trait EI and more social/interpersonal facets in a measure of trait social intelligence.

## Present study

This study will examine the utility of the psychometric method described in the introduction and illustrate its application. Specifically, the method will be applied to the construct of trait EI, as operationalized through the Trait Emotional Intelligence Questionnaire (TEIQue; Petrides, 2009). The TEIQue was designed to assess the construct of trait EI comprehensively and has hitherto produced very promising results in terms of construct validity (Freudenthaler, Neubauer, Gabler, Scherl, & Rindermann, 2008; Gardner & Qualter, 2010; Martins et al., 2010). Its theoretical set of 15 facets was determined through a content analysis of existing measures, retaining only those facets that were common across salient EI models. This unique approach captured the consensus among the existing models and measures, possibly yielding a more accurate representation of the target construct than other models. Evidence attesting that the TEIQue facets satisfy minimum standards for factor loadings has accumulated across translations of the measure (e.g. Freudenthaler et al., 2008; Martskvishvili, Arutinov, & Mestvirishvili, 2013; Mikolajczak, Luminet, Leroy, & Roy, 2007).

Although the model underlying the TEIQue has withstood the test of time, it is possible that some of the numerous facets from which it derives are redundant or extraneous. In this preliminary examination of the proposed method, we used data gathered in previous psychometric studies of the TEIQue, including some of its translations (six samples in total). The data from each sample included measurements of various construct-relevant outcomes. This approach was deemed appropriate for this initial investigation, as the criteria assessed across these samples were diverse and representative of the four TEIQue factors. The principal components from the outcomes assessed in each of the samples were extracted in order to provide alternative representations of global trait EI (Step 2 of the method). These outcome-based composites were then regressed onto the 15 trait EI facets to identify any non-predictive facets. A composite comprising facets with predictive effects in any one or more of the six samples was compared with the original 15-facet composite in terms of its associations with the six criterion-based composites. Facets that did not occupy unique variance in any of the outcome-based composites were further examined to classify them as redundant versus extraneous.

## Method

### Samples and outcomes

The data came from five cross-sectional studies (six samples), in which the criterion validity of the TEIQue across different sets of outcomes was investigated. We selected the samples based on their relevance to the present investigation, as they comprised thematically related, proximate outcomes. Samples 1, 4, and 5 were Greek, Spanish, and Georgian, respectively, whereas Samples 2, 3, and 6 were British. The demographic characteristics of the six samples are summarized in Table1. With the exception of Sample 5, additional details for the samples can be found in previously published studies (Gardner & Qualter, 2010; Petrides, Pérez-González, & Furnham, 2007; Petrides, Pita, et al., 2007).

**Table 1:** Demographic characteristics of samples

Sample (*N*)	Age (years)			Gender	
	*M*	*SD*	Range	Male	Female
1^a^ (271)	25.47	5.88	19–56	92	179
2^b^ (193)	22.83	6.16	18–60	74	118
3^b^ (151)	22.01	6.07	19–54	30	121
4^c^ (202)	23.16	3.35	18–45	35	167
5^d^ (179)	25.58	13.73	17–74	60	117
6^e^ (288)	36.45	11.78	18–79	67	221

*Note*: Samples 1, 4, and 5 were Greek, Spanish, and Georgian, respectively.

a Petrides, Pita, et al., 2007.

b Petrides, Pérez-González, et al., 2007, Study 2.

c Petrides, Pérez-González, et al., 2007, Study 3.

d Martskvishvili, Arutinov, & Mestvirishvili, 2011.

e Gardner & Qualter, 2010.

The outcome variables are presented in Table2, together with their corresponding measures. These outcomes are either entirely emotion-laden (e.g. depression, and positive and negative affect) or integrate emotional and social aspects of functioning (e.g. aggression, coping styles, personality disorders, life satisfaction, alcohol-related problems, and loneliness). Importantly, the outcomes considered across all six samples represent each of the four TEIQue factors (Well-Being, Self-Control, Emotionality, and Sociability), as indicated in Table2. Thus, they are suitable for deriving alternative representations of the trait EI variance, as required in Step 1 of the proposed method.

**Table 2:** Outcome variables and measures used across samples

	Variables	Measures	Trait EI factor represented
Sample 1	Life satisfaction	Satisfaction with Life Scale (Diener et al., 1985)	WB
	Rumination	Emotion Control Questionnaire (Roger & Najarian, 1989)	SC, SOC
	Coping strategies	Coping Styles Questionnaire (Roger et al., 1993)	SC, EMO, SOC
Sample 2	Coping strategies	Coping Styles Questionnaire (Roger et al., 1993)	SC, EMO, SOC
	Depressive symptomatology	Center for Epidemiologic Studies Depression Scale (Radloff, 1977)	WB, EMO
	Depressogenic attitudes and beliefs	Dysfunctional Attitudes Scale (Weissman & Beck, 1978)	WB, EMO
Sample 3	Aggression types	Aggression Questionnaire (Buss & Perry, 1992)	SC, EMO, SOC
Sample 4	Positive and negative affectivity	Positive and Negative Affect Schedule (Sandín et al., 1999; Watson et al., 1988)	WB, SC, EMO, SOC
	General depression	Beck Depression Inventory (2nd ed.; Beck et al., 1996; Sanz, Perdigón, & Vázquez, 2003)	WB, EMO
	Personality disorders	International Personality Disorder Examination (López-Ibor Aliño et al., 1996; Loranger et al., 1997)	WB, SC, EMO, SOC
Sample 5	General depression	Beck Depression Inventory (1st ed.; Beck et al., 1961)	WB, EMO
	State and trait anxiety	State-Trait Anxiety Inventory (Spielberger et al., 1983)	WB, EMO, SOC
Sample 6	Aggression types	Aggression Questionnaire (Buss & Perry, 1992)	SC, EMO, SOC
	Social and emotional (family and romantic) loneliness	Social and Emotional Loneliness Scale for Adults—Short form (DiTommaso et al., 2004)	EMO, SOC
	Eating-related problems	Eating Disorders Diagnostic Scale (Stice et al., 2000)	WB, SC, EMO
	Alcohol-related problems	Self-Administered Alcoholism Screening Test (Hurt et al., 1980)	WB, SC, EMO
	Subjective happiness	Subjective Happiness Scale (Lyubomirsky & Lepper, 1999)	WB
	Life satisfaction	Satisfaction with Life Scale (Diener et al., 1985)	WB

*Note*: Sample 1 measures were administered in Greek, Sample 4 measures in Spanish, and Sample 5 measures in Georgian. EI, emotional intelligence; WB, Well-Being; SC, Self-Control; EMO, Emotionality; SOC, Sociability.

### Measures

All measures in this study were based on self-report, mostly using multiple-point response scales.

#### Trait emotional intelligence

The full form of the TEIQue, which yields global, factor (4), and facet (15) scores, was administered to all six samples. Samples 1–4 completed the initial version (v. 1.00, 144 items), whereas Samples 5 and 6 completed the current version (v. 1.50, 153 items). Samples 2, 3, and 6 completed the TEIQue in its original language (English), whereas Greek, Spanish, and Georgian translations were administered to Samples 1, 4, and 5, respectively. The TEIQue was translated by the researchers who conducted the studies (Martskvishvili et al., 2013; Petrides, Pérez-González, et al., 2007; Petrides, Pita, et al., 2007).

The four factors and their constituent facets are Well-Being (self-esteem, trait happiness, and trait optimism), Self-Control (emotion regulation, stress management, and low impulsiveness), Emotionality (emotion perception, trait empathy, emotion expression, and relationships), and Sociability (assertiveness, emotion management, and social awareness). Two facets (adaptability and self-motivation) have not been included in any of the four factors but contribute directly to the global score. More detailed descriptions of the facets and factors can be found in Petrides (2009). The TEIQue items are responded to on a 7-point Likert scale, ranging from 1 (*disagree completely*) to 7 (*agree completely*). Internal consistencies at the facet level were predominantly within a range of .70 to .80 across studies. Cronbach's alphas for global trait EI ranged from .81 (Sample 5) to .96 (Sample 6).

#### Outcome variables

A summary of the outcome measures and references can be found in Table2. The measures administered to Sample 1 were translated by the authors who conducted the study. For Samples 4 and 5, the outcomes were assessed with available translations of the measures.

##### Sample 1

The Satisfaction with Life Scale (Diener, Emmons, Larsen, & Griffin, 1985) consists of five items that yield a global life satisfaction score (e.g. ‘In most ways my life is close to my ideal’) measured on a 7-point Likert scale. Cronbach's alpha in this sample was .84.

The 14-item rehearsal subscale from the Emotion Control Questionnaire (Roger & Najarian, 1989) was used as a measure of rumination (e.g. ‘I remember things that upset me or make me angry for a long time afterwards’). Items are responded to on a 7-point Likert scale. Cronbach's alpha was .84.

The Coping Styles Questionnaire (Roger, Jarvis, & Najarian, 1993) consists of 60 items assessing four coping strategies. Two of these (rational and detached coping) are considered to be adaptive, and the other two (emotional and avoidant coping), maladaptive. Items are responded to on a 4-point Likert scale. Cronbach's alphas were .81 (rational coping), .80 (detached coping), .84 (emotional coping), and .68 (avoidant coping).

##### Sample 2

Sample 1 completed a Greek translation of the Coping Styles Questionnaire, while Sample 2 completed the original English version. Cronbach's alphas were .82 (rational coping), .84 (detached coping), .83 (emotional coping), and .68 (avoidant coping).

The Center for Epidemiologic Studies Depression Scale (Radloff, 1977) is a 20-item measure of depressive symptomatology, specifically developed for use in non-clinical settings. Respondents indicate how frequently they experience a range of depressive symptoms during the past week (e.g. ‘I was bothered by things that usually don't bother me’). Items are responded to on a 4-point Likert scale. Cronbach's alpha was .92.

The Dysfunctional Attitudes Scale (Weissman & Beck, 1978) is a measure of depressogenic attitudes and beliefs, based on a cognitive theory perspective and consisting of two parallel 40-item forms. Using a 7-point Likert scale, respondents answer each item according to how they think most of the time. Form A was administered to Sample 2, yielding an alpha level of .87.

##### Sample 3

The Aggression Questionnaire (Buss & Perry, 1992) assesses four distinct types of aggression. It consists of 29 items responded to on a 5-point Likert scale. The four aggression scales, and their respective internal consistencies, are physical aggression (.80), verbal aggression (.69), anger (.80), and hostility (.79).

##### Sample 4

The Positive and Negative Affect Schedule (Sandín et al., 1999; Watson, Clark, & Tellegen, 1988) was used to assess positive and negative affect. Each affective dimension has 10 items that are responded to on a 5-point Likert scale. The alpha level was .89 for positive affect and .85 for negative affect.

The second edition of the Beck Depression Inventory (Beck, Steer, & Brown, 1996; Sanz, Perdigón, & Vázquez, 2003) was administered to this sample. It measures the severity of depression and consists of 21 items that are responded to on a 4-point Likert scale. The alpha level was .87.

The International Personality Disorder Examination (López-Ibor Aliño, Pérez Urdaníz, & Rubio Larrosa, 1996; Loranger, Janca, & Sartorius, 1997) has a semi-structured interview format aligned to the ICD-10 and DSM-IV criteria. Typically used as a screener, this instrument comprises 77 dichotomous true-or-false items that produce scores representative of 10 distinct personality disorders. Alpha levels were generally low to moderate, ranging from .32 for Schizoid to .67 for Avoidant.

##### Sample 5

The first edition of the Beck Depression Inventory (Beck, Ward, Mendelson, Mock, & Erbaugh, 1961) was administered to Sample 5. Like its successor, which was administered to Sample 4, this edition measures the severity of depression and consists of 21 items that are responded to on a 4-point Likert scale. The alpha level was .81.

The State-Trait Anxiety Inventory (Spielberger, Gorsuch, Lushene, Vagg, & Jacobs, 1983) comprises 40 items, which are based on a 4-point Likert scale and represent two types of anxiety: state and trait anxiety. Accordingly, scores can be derived for both state and trait anxiety, which had alpha levels of .85 and .81, respectively.

##### Sample 6

The Aggression Questionnaire (Buss & Perry, 1992), as described in Sample 3, was also administered to this sample. The internal consistencies were .71 for physical aggression, .65 for verbal aggression, .66 for anger, and .69 for hostility.

The Social and Emotional Loneliness Scale for Adults—Short Form (DiTommaso, Brannen, & Best, 2004) contains 15 items that are responded to on a 7-point Likert scale. The items are evenly distributed across three subscales assessing family loneliness (α = .89), romantic loneliness (α = .96), and social loneliness (α = .89).

The Eating Disorders Diagnostic Scale (Stice, Telch, & Rizvi, 2000) consists of 22 items, 19 of which (items 1–18 and 21) are used to derive the single composite of this scale. One of the 19 items (item 21, addressing amenorrhea) was omitted in order to make the scale suitable for participants of both genders. The measure's items have a mix of Likert-type and *yes*-or-*no* response formats. In this sample, the internal consistency was .86.

The Self-Administered Alcoholism Screening Test (Hurt, Morse, & Swenson, 1980) consists of 35 dichotomous *yes*-or-*no* items, indicative of alcohol-related problems. Its internal consistency in this sample was .76.

The Subjective Happiness Scale (Lyubomirsky & Lepper, 1999) consists of four items that are responded to on a 7-point Likert scale. Its internal consistency in this sample was .89.

The Satisfaction with Life Scale (Diener et al., 1985) previously described in Sample 1 was also administered to this sample, in which it had an alpha level of .90.

### Statistical analyses

The outcome variables corresponding to each sample were submitted to a principal component analysis to derive the outcome-based composites. Outcome variables were included within the respective outcome-based composite if they had loadings either (a) in excess of .50 or (b) of .30–.49 that were greater than their loadings on ensuing components. Conversely, variables were excluded from the analyses if they loaded weakly on the first principal component (<.50) and more strongly on ensuing components. These variables were deemed to be too distinct from the target construct, with additional dimensions implicit in them increasing the chances of predictive effects for ET facets (or for the specific variance of RD facets).

The derived outcome-based composites were regressed onto the 15 trait EI facets, using the stepwise method in each analysis. All facets were entered at the first step and subsequently removed successively, starting with the least significant one. Because the stepwise method was used, as required by the method, it was possible for facets already removed to be re-entered at later steps of the analyses.

The original composite of all 15 trait EI facets and a composite comprising facets included in the final model in at least one of the six regression analyses were compared in terms of their associations with the outcome-based composites. Facets with significant predictive effects in any of the six samples were included in this composite to account for variations in the outcomes used to derive the outcome-based composites. Steiger's *Z* tests were computed to examine if there are significant differences in the correlations of these two composites with the outcome-based composites across samples.

To differentiate between RD and ET facets, zero-order correlations of any non-predictive facets with a revised composite comprising the predictive facets only were also examined. In theory, RD facets should correlate significantly with the global construct, whereas ET facets should show correlations closer to zero.

## Results

### Dimension reduction of outcome variables

Results of the principal component analyses for the outcomes used in each sample are presented in Table3. The only variable excluded from Samples 1 and 2 was avoidance coping because it had relatively weak loadings (.14 and −.46, respectively) on the first principal component. It also resulted in bifactorial solutions in the initial analyses, loading considerably higher on the second component. For the same reasons, three personality disorders were removed from the final analysis of the Sample 4 outcomes: schizoid, histrionic, and narcissistic. Their respective loadings on the first principal component were .38, .36, and .24, and lower than their loadings on a second or third component. Two variables, verbal aggression and eating-related problems, were excluded from the Sample 6 outcomes. Their loadings on the first principal component were .32 and .27, respectively, and both loaded much higher on additional components. These seven variables were excluded on the grounds that they were too different from the target construct. With these variables omitted, a latent composite was derived from the remaining variables in Samples 1, 2, 4, and 6. All outcome variables assessed in Samples 3 and 5 were included in their respective composites, as they all loaded highly on a single principal component.

**Table 3:** First principal component loadings for sample outcomes

	Variable	Factor loading	Communality	% of variance
Sample 1	Life satisfaction	.63	.40	51.87
	Rumination	.59	.35	
	Rational coping	.78	.61	
	Detached coping	.80	.64	
	Emotional coping	−.77	.59	
Sample 2	Rational coping	.77	.59	55.37
	Detached coping	.77	.59	
	Emotional coping	−.83	.70	
	Depressogenic attitudes and beliefs	.55	.30	
	Depressive symptomatology	.77	.59	
Sample 3	Physical aggression	.73	.53	52.39
	Verbal aggression	.63	.39	
	Anger	.86	.73	
	Hostility	.66	.44	
Sample 4	IPDE paranoid	.73	.58	44.42
	IPDE schizotypal	.76	.62	
	IPDE antisocial	.52	.62	
	IPDE borderline	.78	.61	
	IPDE obsessive–compulsive	.48	.32	
	IPDE dependent	.58	.41	
	IPDE avoidant	.68	.47	
	Negative affect	.73	.54	
	Positive affect	−.53	.61	
	General depression	.78	.65	
Sample 5	Depression	.83	.68	74.42
	State anxiety	.89	.79	
	Trait anxiety	.87	.76	
Sample 6	Physical aggression	.44	.61	40.53
	Anger	.53	.71	
	Hostility	.75	.61	
	Social loneliness	.62	.52	
	Family loneliness	.63	.56	
	Romantic loneliness	.58	.45	
	Alcohol-related problems	.37	.23	
	Subjective happiness	−.80	.65	
	Life satisfaction	−.83	.72	

*Note*: Avoidance coping was excluded from Samples 1 and 2, as it loaded relatively weakly on the first principal component and more strongly on a second component. For the same reason, the IPDE schizoid, histrionic, and narcissistic scales were excluded from Sample 4, and verbal aggression and eating-related problems from Sample 6. IPDE, International Personality Disorder Examination (Loranger et al., 1997).

### Regression of outcome-based composites on trait emotional intelligence facets

Summaries of the stepwise regression analyses with the outcome-based composites as the dependent variables are presented in Table4. Because of the large amount of data, we present only results for the initial and final models and beta weights for facets retained in the final model only. While all 15 facets were initially included in the analyses, facets that were not retained in the last step of any of the six regression models are omitted from Table4. The analyses for Samples 3, 4, and 6 excluded the facet of emotion management, while that for Sample 6 additionally excluded the facets of trait empathy and emotion perception. The reason for omitting these facets is that when initially included, the direction of their explanatory effect was opposite to those of the other facets in the equations. Full results can be requested from the authors.

**Table 4:** Stepwise regression summaries for trait emotional intelligence facets predicting the outcome-based composites

Trait EI facets	Sample 1		Sample 2		Sample 3		Sample 4		Sample 5		Sample 6	
	β	*R*^2^_Adj_	β	*R*^2^_Adj_	β	*R*^2^_Adj_	β	*R*^2^_Adj_	β	*R*^2^_Adj_	β	*R*^2^_Adj_
Model 1 (all facets)		.68		.72		.37		.59		.54		.77
Final model		.67		.72		.38		.58		.54		.76
Self-motivation											−.10**	
Emotion regulation	−.20***		−.20***		−.21**							
Trait happiness					−.24**		−.29***		−.25***		−.58***	
Low impulsiveness					−.19*		−.12*				−.11**	
Self-esteem	−.20***		−.20**						−.31***			
Assertiveness			−.14**				−.17***					
Trait optimism	−.30***		−.27***									
Relationships					−.26***		−.21***				−.21***	
Adaptability	−.12*						−.13*					
Stress management	−.24***		−.33***				−.18**		−.36***		−.10**	
Δ*R*^2^		−.02		−.01		−.03		−.02		−.01		−.00
*N*		271		193		151		202		179		288

*Note*: Only beta weights for facets retained in the final models are displayed. EI, emotional intelligence.

* *p* < .05.

** *p* < .01.

*** *p* < .001.

Of the 15 trait EI facets, five did not explain unique variance in the outcome-based composites in any sample and, thus, do not appear in the final regression models. These facets were trait empathy, emotion perception, emotion expression, emotion management, and social awareness. In addition to being manually excluded from Samples 3, 4, and 6, emotion management did not appear in the final regression models in Samples 1, 2, and 5, based on the stepwise method. Likewise, trait empathy and emotion perception, which were manually removed from the Sample 6 regression, were non-predictive in the other samples. Therefore, neither these three facets nor the two non-predictive facets appear in Table4. Of the 10 facets showing significant predictive effects, one (stress management) accounted for unique variance in five samples, one (trait happiness) accounted for unique variance in four samples, four (emotion regulation, self-esteem, impulsiveness, and relationships) accounted for unique variance in three samples, two accounted for unique variance in two samples (assertiveness and trait optimism), and two, self-motivation and adaptability, accounted for unique variance in one sample.

In comparing the additive predictive effects of all 15 facets included in the initial prediction model (shown as Model 1) against the final set of facets remaining in the last step of each regression analysis (shown as Final model), the appropriate statistic to examine is the adjusted *R*^2^, which can account for the unequal degrees of freedom. As is apparent across all six samples, the shortened sets accounted for virtually the same amount of the variance as the 15-facet composite. Even the unadjusted change in *R*^2^ from the initial to final model was negligible and non-significant in the six samples. As discussed, however, regression analysis does not reveal the impact of non-predictive facets or facets with atheoretical, inverted effects on the explanatory power of higher-order composites, such as global trait EI. For example, the non-predictive facets of emotion expression and trait empathy can be expected to weaken the convergence of global trait EI with the outcome-based composites, because they are averaged along with the predictive facets into the global trait EI score. Hence, two trait EI composites comprising 15 and 10 facets, respectively, were compared in terms of their associations with the outcome-based composites.

### Criterion validity of facet-based composites

Pearson correlations of the 15-facet and 10-facet trait EI composites with the outcome-based composites are presented in Table5. Also shown are Steiger *Z* tests of significant differences in the convergent validity of the two composites. Except for the latent composite derived from the Sample 3 outcomes, associations of both trait EI composites with the outcome-based composites were consistently strong. Unlike the other samples, in which a latent composite of more diverse emotion-related outcomes was used, the outcome-based composite derived from the aggression variables in Sample 3 was fairly homogenous and narrow and, thus, least representative of trait EI. Correlations of the 10-facet composite with the outcome-based composites were consistently larger than those of the 15-facet composite. In fact, the Steiger *Z* results indicate that the 10-facet composite had significantly greater convergent validity in all six samples.

**Table 5:** Correlations of the 15-facet and 10-facet trait emotional intelligence composites with the outcome-based composites

Sample (*N*)	15-facet composite	10-facet composite	Steiger's *Z*
1 (271)	.73	.79	4.94**
2 (193)	−.75	−.80	3.88**
3 (151)	−.49	−.58	3.79**
4 (202)	−.73	−.76	2.34*
5 (179)	−.65	−.68	2.27*
6 (288)	−.78	−.81	3.10**

*Note*: All correlations are significant at *p* < .001.

* *p* < .05.

** *p* < .01.

### Correlations of non-predictive facets with 10-facet composite

Correlations between the five non-predictive facets and the 10-facet composite are shown in Table6. All correlations were significant, and all except one (emotion management in Sample 3) were within a moderate range of .3 to .7, indicating that the facets are redundant, rather than extraneous.

**Table 6:** Correlations of the five non-predictive trait emotional intelligence facets with the 10-facet composite

Sample (*N*)	Trait empathy	Emotion perception	Emotion expression	Emotion management	Social awareness
1 (271)	.32	.51	.38	.43	.66
2 (193)	.34	.48	.52	.46	.70
3 (151)	.35	.49	.50	.21*	.63
4 (202)	.46	.57	.40	.32	.64
5 (179)	.36	.52	.44	.36	.54
6 (288)	.36	.47	.48	.32	.57

*Note*: Correlations not denoted by an asterisk are significant at *p* < .001.

* *p* < .01.

## Discussion

Decades ago, Cronbach and Meehl (1955) noted that there is no adequate criterion for operationally defining personality traits and other psychological constructs, which prompted their concept of construct validity. In the present day, researchers continue to dwell on the level of arbitrariness involved in facet selection (e.g. Petrides & Furnham, 2001). The psychometric method illustrated herein is an effort towards optimizing multi-faceted assessment instruments, including the construct representations on which they are based. As specified throughout the article, its particular aim is to identify RD and ET facets. The method thereby aims to reduce the plethora of facets through which constructs are often represented and to minimize discrepancies between assessment instruments.

### Summary and interpretation of results

Application of the method to trait EI data from six European samples yielded consistent results. Five facets did not explain unique variance in alternative representations of the construct variance, derived from varying sets of validation outcomes administered across the six samples. Removal of these five facets from the global trait EI composite significantly improved its associations with the outcome-based composites in all samples. Collectively, the results indicate that the five non-predictive facets overlap entirely with the predictive facets in their reliable common variance (i.e. variance attributed to the construct of trait EI), apparently compromising the construct validity of the global trait EI composite. It seems that the revised 10-facet composite gives a better representation of trait EI than the original composite.

The trait EI facets identified as non-predictive came exclusively from the TEIQue factors of Emotionality and Sociability. Notably, these two factors have shown little success in explaining incremental criterion variance vis-à-vis the other factors in previous research (Mikolajczak, Luminet, & Menil, 2006; Mikolajczak, Roy, Verstrynge, & Luminet, 2009; Mikolajczak et al., 2007; Swami, Begum, & Petrides, 2010; Uva et al., 2010; Siegling, Vesely, Petrides, & Saklofske, accepted). In only one study, one of these two subscales (Sociability) accounted for incremental criterion variance, predicting somatic symptoms amid stress over mental and physical status, together with the Self-Control subscale (Mikolajczak et al., 2006). However, it is important to remember that individual criteria are unlikely to represent the variance of the target construct very well, and therefore, significant predictive effects of redundant and extraneous elements are possible.

While all of the Self-Control and Well-Being facets explained incremental variance in the expected direction in at least one of the samples of the present study, the Sociability and Emotionality factors had only a single facet each that occupied variance in at least one of the outcome-based composites. Zero-order correlations of the non-predictive facets with the 10-facet composite were within a moderate range and significant, suggesting that the identified facets are redundant.

A shared characteristic of the five non-predictive facets is their integration of interpersonal emotional attributes, although some merge interpersonal and intrapersonal qualities (e.g. emotion perception represents the propensity to perceive emotions in oneself and in others). This pattern is consistent with some evidence speaking to the distinctiveness of these types of facets (Siegling, Saklofske, Vesely, & Nordstokke, 2012; Siegling, Vesely, & Saklofske, 2013). As discussed previously (Siegling et al., 2012, 2013), it is possible that some of these facets (e.g. emotion management of others and trait empathy) share most of their variance with constructs more indicative of social behaviour, such as trait social intelligence (Petrides et al., 2011).

Although a similar set of predictive facets is likely to emerge in independent samples and across different outcome-based composites, fluctuations in terms of which facets will have significant effects are still possible. A statistical factor to consider is that facets may emerge as significant or non-significant because of chance. Self-motivation may be such a candidate, as it had a significant incremental effect in only one of the six samples and the regression weight for its effect was very small. Although a scenario of all five presumably RD facets being unrepresented in the outcome variables is highly unlikely, it is also possible that some segments of the construct variance were not represented in the outcomes we investigated. Consequently, facets related to any under-represented construct variance would not have reached significance. While we do not expect large fluctuations in the pattern of predictive facets, repeated applications of the method to trait EI data are encouraged to increase confidence in our findings. It is also important to validate the revised composite in independent samples and sets of criteria that have not been previously used to identify non-predictive facets.

Empirical characteristics of RD and ET facets are failing to occupy unique construct variance and compromising the construct validity of the global composite. RD facets share the same common variance with one or more of the other facets, giving disproportional weight to particular segments of the construct variance. ET facets lie wholly beyond the target construct's boundaries, thus lacking common variance (i.e. their variance is due to constructs other than the one targeted). Neither of these types of facet is, therefore, able to take up unique variance in the global construct, thus weakening the construct validity of the model that incorporates them and of its operational vehicles. Overall, the results provide preliminary evidence for the efficacy of the proposed method in identifying RD facets, because all of the non-predictive facets seemed to fall into this category. At least in theory, it should also screen out facets that are completely extraneous and somehow found their way into the researcher's model.

### Implications of method

Subject to further validation, the method has utility in the optimization of multi-faceted assessment instruments. As discussed, a unique strength of the proposed strategy lies in its potential to identify RD or ET facets, which conventional approaches do not accomplish. Identification and eventual removal of RD and ET facets would help improve the construct validity of measures to which the method is applied. Similarly, the method has promise in enhancing the unidimensionality or homogeneity of scales intended to assess a single construct, the importance of which has been discussed in detail elsewhere (Smith, McCarthy, & Zapolski, 2009; Smith & Zapolski, 2009). On a larger scale, the method would contribute to minimizing the inflation of facets and diversification of measures.

Beyond minimizing research costs, optimizing the scale-construction process by integrating this method can lead to more valid conclusions about constructs, especially at the earlier stages of research. Without applying the method, a model or measure may comprise ET or RD facets and, thus, have weaker construct validity. Naturally, these facets would also compromise the various specific and empirically testable aspects of construct validity (criterion, predictive, discriminant, etc.) pertaining to the measure or model being scrutinized. By applying the method first in order to gain construct validity, it would be possible to assess and understand the construct's relationships with other constructs and outcomes more accurately.

If thoroughly applied, the method would entail realistic benefits for psychology's applications, particularly where quantitative assessment is involved. On a general level, it would enhance the professional and social utility of a range of standardized measures, enabling more accurate assessments of individuals and prediction of their future behaviour. Failing to represent and measure a construct adequately can have consequences, given that psychometric assessment often forms the basis of high-stakes decisions, such as clinical diagnoses, career selection, and people matching. Another benefit of identifying, and eventually removing, RD and ET facets is the reduced length of psychometric measures and shorter assessment times without trade-offs (Smith et al., 2003). In view of these benefits, the method would be ideally integrated at the early stages of scale construction. For constructs that already have an established operationalization, the method can be used either to refine these measures or, should non-predictive facets not emerge, to increase confidence in them and their underlying models.

### Recommendations and projected developments

Particularly when used to construct a new measure, the method should be applied in combination with the established methods, of which one (the rational–theoretical approach) is even a pre-requisite. Furthermore, as indicated throughout the article, it may be wisest to consider the method as an ongoing process, whereby repeated application across samples of participants and outcomes will increase certainty in the identification of RD and ET facets. Beyond the method *per se* (described here as a five-step procedure), another worthwhile step would be to cross-validate the results, by comparing the revised and original composites in samples of criteria not used during application of the method.

Future developments of the method are foreseeable with regard to two of its five steps. The first concerns the process of selecting and testing outcomes for deriving alternative representations of the construct variance at Steps 1 and 2. We anticipate that with theoretical development and repeated application of the method, more specific examples and guidelines for outcome selection will emerge. Second, while the statistical procedures employed in this article (particularly at Step 3) can identify RD and ET facets, they are of limited utility in examining the relative proportions that the remaining facets occupy within the construct variance, because of intercorrelations among predictors. However, new approaches, such as relative weight analysis (Johnson, 2000), may be able to estimate the relative common variances occupied by facets at Step 3. This information would provide insight into the centrality of the different valid facets and further our understanding and conceptualization of the construct. Last, while the generic problems associated with stepwise regression algorithms are of lesser threat to the proposed method, given its multi-sample and replication requirements, additional adjustments may be reasonable (e.g. accounting for chance effects by using different *p*-value cut-offs or effect size estimates).

### Limitations and future directions

Further validation of the proposed method with respect to other personality constructs is needed to provide definitive evidence for its efficacy. Once a satisfactory level of support has been established, it would be worthwhile to demonstrate that the method also has efficacy within the realm of cognitive abilities, as can be expected. Whereas this article presents the initial application of the method, based on existing data, future studies designed specifically for its evaluation can yield more conclusive results. However, this is not to undermine the utility and relevance of using existing datasets, as the method requires evidence from numerous and relatively large samples. We encourage others who have suitable data (ideally, from multiple samples) to replicate the analyses we performed here and publish the results.

In designing future studies specifically for applying the proposed method, it will be important to sample systematically from the entire theoretical range of relevant outcomes to represent the variance of the target construct as comprehensively as possible. A second question to be addressed in further validation studies of the method is whether using the same measurement format for all variables introduces confounding effects in favour of the method. Measuring the outcomes in the same way as the hypothetical facets creates common-method variance (e.g. social desirability), which may contribute to the pattern of results. Alternative methods (i.e. other than self-report) for assessing outcomes relevant to trait EI and other personality constructs include informant ratings, behavioural observations, electronic diaries, and possibly biodata. Converging evidence from applications of the method across various outcome-based composites will eventually help us arrive at a consensus regarding the best set of facets for representing established, yet still partially elusive, individual-differences constructs.
